# Academia’s Big Five: a normative taxonomy for the epistemic responsibilities of universities

**DOI:** 10.12688/f1000research.19459.2

**Published:** 2020-07-06

**Authors:** Rik Peels, René van Woudenberg, Jeroen de Ridder, Lex Bouter

**Affiliations:** 1 Philosophy Department, Vrije Universiteit Amsterdam, Amsterdam, The Netherlands; 2 Amsterdam Medical Centers, Amsterdam, The Netherlands

**Keywords:** Big question, epistemic responsibilities, humanities, research integrity, societal relevance, teaching, university, virtue

## Abstract

This paper proposes a normative taxonomy by which universities can express the extent to which they meet five core epistemic responsibilities. Epistemic responsibilities are responsibilities that have to do with the attainment of knowledge and understanding. The core epistemic responsibilities, which we call the Big Five, are to (1) foster research integrity, (2) teach for intellectual virtue, (3) address the big questions of life, (4) give humanistic inquiry and education a proper place, and (5) serve society. The paper characterizes the Big Five in some detail and explains why they are core epistemic responsibilities of universities. The paper concludes by describing the steps that should be taken in order to test, amend, and implement the taxonomy.

## Introduction

In this paper, we propose a normative taxonomy of what we call the ‘Big Five’ in academia: five core epistemic responsibilities of universities. Epistemic responsibilities are responsibilities that have to do with the attainment of knowledge, understanding, insight, rationality, and explanation. This is not to deny that universities have other important responsibilities. Among them are moral responsibilities (like providing safe environments for students and faculty, and taking care of the wellbeing of human and animal test subjects, treating native people fairly); legal responsibilities; financial responsibilities; social responsibilities (like producing useful technologies and effective medical interventions), and more ^1^. However, *all* institutions and companies have various responsibilities of these kinds, not only universities. What sets universities apart from many other organizations and institutions is that their main goals are *epistemic* in nature: to produce knowledge, to understand, to gain insight into phenomena, and so on. That is why we focus on epistemic responsibilities in this paper.

For each epistemic responsibility, we distinguish five levels describing the extent to which a university meets that responsibility or strives to do so. Our proposed taxonomy is meant as a tool to assess the degree to which a university meets its core epistemic responsibilities. The format we use is inspired by the Transparency and Openness Promotion (TOP) guidelines ^2^ that journals can use to describe the extent to which they meet the goals of Open Science.

The taxonomy proposed here is a product of two research projects funded by the Templeton World Charity Foundation: *Science beyond Scientism* (2013–2016) and *The Epistemic Responsibilities of the University* (2016–2019) ^3^. The first project explored which questions science can and which ones it cannot address, as well as whether natural sciences are the only reliable source of knowledge. The second project explored what the core epistemic responsibilities of universities are, given various contemporary challenges, such as hypercompetition, publication pressure, the marginalization of the humanities, and the commercialization of the university ^4^. In both projects, philosophers worked in close cooperation with biomedical and social scientists. We present this as work in progress, as a starting point for gaining experience with using the taxonomy and consensus building for a more mature version. We will do so in a third project entitled *Epistemic Progress in the University (2020–2023)* that was recently funded by the Templeton World Charity Foundation.

This paper is structured as follows. First, we provide our proposed taxonomy by specifying academia’s Big Five epistemic responsibilities and detailing five levels of meeting them (§2). After that, we argue that these are indeed five core epistemic responsibilities of universities (§3). Finally, we lay out which future steps we aim to take to test, amend, and implement the taxonomy (§4).

## A normative taxonomy

Our proposal distinguishes five epistemic responsibilities (see Table 1). We assume each to be of more or less equal importance, so the order in which we present them is not hierarchical. The attainment levels I through V for each responsibility, however, *are* meant hierarchically: each level presents a more advanced stage of meeting the responsibility at issue. We think of these responsibilities as attaching primarily to entire universities. So, in order to meet them, each responsibility should have consequences systematically, throughout the university and its faculties, departments, institutes, or other organizational parts. If a university strives to teach for intellectual virtue at the highest level, for example, students throughout the university should be instructed in what these virtues are and stimulated to cultivate them through virtue-building teaching activities.

**Table 1.** Normative taxonomy of epistemic responsibilities of universities.

Big Five Epistemic Responsibilities	Level I	Level II	Level III	Level IV	Level V
**1. To foster research** **integrity**	Issues of detrimental research practices and responsible conduct of research are neglected	Issues of detrimental research practices and responsible conduct of research are occasionally addressed	Detrimental research practices are addressed in response to breaches and problems, but there is no academic climate that actively stimulates responsible conduct of research	There is an academic climate that detects and acts upon detrimental research practices and actively stimulates responsible conduct of research, but only at the level of individual researchers	There is an academic climate that detects and acts upon detrimental research practices and actively stimulates responsible conduct of research, at both the level of individual researchers and that of teams and departments
**2. To teach for** **intellectual virtue**	The university teaches for knowledge and understanding, but pays no attention to intellectual virtues	The university teaches for knowledge and understanding, and intellectual virtues are considered important but not taught	The university teaches for knowledge and understanding and includes education about what the intellectual virtues are	The university teaches for knowledge and understanding, explicitly includes intellectual virtues in education, and explores cases that show the relevance of intellectual virtues	The university teaches for knowledge and understanding, includes intellectual virtues in education, and practically engages in developing intellectual virtues
**3. To address the big** **questions of life**	The big questions are neglected; there is no attention for them	The big questions are mentioned, but discarded as meaningless or outside the scope of universities	The big questions are taken seriously and addressed in teaching, but in isolation, within relatively isolated departments or courses	The big questions are taken seriously and addressed in (interdisciplinary) research and teaching in some departments and courses	The big questions are taken seriously and addressed throughout the university’s (interdisciplinary) research and teaching in all programs and departments
**4. To give humanistic** **inquiry and education a** **proper place**	The humanities are acknowledged as legitimate academic disciplines, but marginalized	The humanities are not marginalized, but considered and treated as inferior to the sciences	The humanities have a proper place in the university, but operate in isolation	The humanities have a proper place in the university, and there is cooperation with some other disciplines in teaching and research	The humanities have a proper place in the university and there is intensive and broad cooperation across disciplines in teaching and research
**5. To serve society**	Research and teaching is confined to what is considered relevant for purely academic challenges	Research and teaching identifies societal challenges, but the university leaves it to others to confer knowledge and understanding relevant to those challenges	Research and teaching that addresses societal challenges is carried out upon request, but deemed substantially less important than fundamental research and teaching	Research and teaching that addresses societal challenges has a fixed place in the university, but is deemed less important than fundamental research and teaching	Research and teaching that addresses societal challenges has a fixed place in many departments and programs, and is seen as equally important as fundamental research and teaching

This doesn’t mean that there cannot be an internal division of labor when it comes to meeting epistemic responsibilities: giving humanistic inquiry a proper place will primarily fall on humanities departments, although humanities scholars will teach in other departments and collaborate with scientists in other departments as well when a university strives for level V of this responsibility. Similarly, not all departments and research teams will have to serve society in the same way or to an equal degree. There will be significant differences between, say, the theoretical physicists and the nutrition scientists.

We will now clarify each responsibility briefly and motivate why it belongs on the list of epistemic responsibilities of universities.

**1. *To foster research integrity*.** Research integrity is fostered by getting rid of perverse incentives, stimulating good mentoring, having an open research climate, and so on. Detrimental research practices include both rare major research misbehaviors like fabrication of data and highly prevalent minor misbehaviors like selective reporting ^5^. By ‘responsible conduct of research’ we mean behavior that meets the principles and standards for good research, as laid out in major codes of conduct for research integrity ^6^. This can be done on the level of individual scholars, but also that of *groups*, such as research teams or departments. Ideally, research integrity is promoted for both individuals and groups.

The results of scientific and scholarly research play a crucial role in modern society. Universities carry out a substantial part of this research and educate and train researchers who perform the studies and apply the results. To ensure the validity and trustworthiness of findings research needs to be performed according to the principles and standards for research integrity. In recent years it has become painfully evident that there is substantial room for improvement in the level of compliance to these principles and standards ^7^. Surveys indicate that 2% of researchers admit to having fabricated or falsified data themselves, while one third says that they have been engaged in less serious questionable research practices ^8^. Only 10 – 40% of study results turn out to be reproducible when the study is repeated ^9^. This is often due to small sample sizes, selective reporting, and other questionable research practices ^10^. Although these phenomena are understudied, it is to be expected that more transparency ^11^ and specifically preregistration ^12^ of study protocols and data analysis plans will lead to improvements. This epistemic responsibility entails not only the adoption of transparency and educating staff and students in responsible conduct of research, but also removing perverse incentives from the ways in which researchers are assessed ^13^ and performing research on research to strengthen the evidence base for optimizing research integrity.

**2. *To teach for intellectual virtue*.** Among the intellectual virtues are: openmindedness, attentiveness, charitableness, intellectual courage, creativity, curiosity, discernment, honesty, intellectual humility, objectivity, parsimony, perseverance, studiousness, wisdom ^14^. Educating for intellectual virtues is part of the traditional ideal of *Bildung*. Moral virtues, such as generosity, kindness, or benevolence, may well also be important and universities might have a responsibility to cultivate them, but, if so, this isn’t an *epistemic* responsibility. When universities choose to teach for intellectual virtues, they can do so, minimally, by merely bringing up these virtues in educational settings; or they can explore them through case studies of intellectually virtuous scientists and scholars and their contributions to epistemic progress, or, most advanced, they can actively cultivate intellectual virtues in their students by having them mimick and *practice* intellectually virtuous behavior. Developing intellectual virtues requires not merely instruction about virtues or reflection on them, but also training and exercise ^15^.

Teaching for intellectual virtues is a responsibility of universities almost by definition. Intellectual virtues are broadly understood as qualities that make someone a well-trained thinker or inquirer ^16^. Hence, the two main tasks of universities, research and teaching, are both served by teaching for intellectual virtue. By aiming to train students to become skilled thinkers (perhaps among other things), universities set an ambitious goal for education. Similarly, academic research also needs skilled thinkers. Moreover, the intellectual virtues are widely relevant outside the university: in politics, journalism, medicine, law, law enforcement, social work – it’s hard to think of any sphere of life were good, analytical, critical, clearheaded, creative, or otherwise high quality thinking wouldn’t matter.

**3. *To address the big questions of life*.** By ‘the big questions’ we mean such questions as: What is the origin and ultimate destination of all that exists? What is the future of the earth’s ecosystem? What is consciousness? Do humans have free will? Is there (objective) good and evil? Can the human mind understand the world and, if so, how? Does life have meaning? Does God exist? How does science relate to religion? These are universal questions in the sense that they have been asked in most cultures and societies throughout history, up to the present; they are not restricted to local concerns or specific academic disciplines.

Addressing the big questions of life is an epistemic responsibility of the university for a number of reasons. First, these questions are too important to be left entirely to non-academics. The big questions are everyone’s concern, academics included. Second, many academics themselves are greatly interested in these questions, even if their specialized science and scholarship do not or even cannot answer them. Third, clarifying and attempting to answer these questions affects how we look at ourselves and what we deem important. Fourth, several big questions are factual questions – highly abstract and large scale, but factual nonetheless. They are not questions about tastes or preferences. It seems like they admit of true or false answers. So, at least in principle, they fall within the purview of scientific and humanistic inquiry. Fifth, big questions can inspire smaller and more manageable research questions. For example, asking about the fundamental nature of reality led to the hypothesis of atomism in Ancient Greece ^17^. Ideas about God’s perfection and omnipotence led Galileo to assume that mathematics is our best guide to understanding the orbits of the planets and hence that heliocentrism rather than geocentrism is correct ^18^. Darwin asked whether humans are unique or whether all life on earth is monogenetic, which led him to develop evolutionary theory ^19^. Einstein opposed the Copenhagen interpretation of quantum mechanics on metaphysical grounds, as he didn’t believe the universe could be fundamentally probabilistic ^20^. For these reasons universities cannot and should not operate as if the big questions don’t exist or cannot be taken seriously. Rather, they should take them seriously and mobilize their intelligence as well as their state of the art science and scholarship to address these questions in an intellectually responsible way.

**4. *To give humanistic inquiry and education a proper place*.** Universities ought to have room for the full range of academic disciplines, in both the sciences and the humanities. Many of today’s most urgent challenges in society cannot be solved by purely scientific or technological means; successful solutions require compelling communication, consideration of moral values and norms, in-depth understanding of cultures and religions. All of these things, and much more, are studied in the humanities. Hence, universities ought to facilitate and embrace humanistic inquiry and teaching, and stimulate interdisciplinary collaborations between scientists and humanities scholars. Of course, some universities – technological universities, for instance – do not include humanities departments. For them, this responsibility may be interpreted as follows: the responsibility to give due weight to knowledge and understanding produced by humanities departments in *other* universities. Of course, this does not remove other epistemic responsibilities that are met primarily in the humanities, such as the epistemic responsibility to address the big questions of life.

Giving the humanities a proper place is a responsibility of the university for one basic reason: the humanities can deliver truth, knowledge and insight in areas where the sciences cannot ^21^. The humanities have their own objects of study: they study objects that have “meaning” in a special sense, viz. *meaning that derives from human conventions, from human intentions, and/or from human purposive behavior* ^22^. The knowledge and understanding the humanities provide differs from the knowledge and understanding that the sciences offer, in that the former is often ‘indexical’ (that is, related to human interests and concerns), ‘perspectival’ (it specifies how things look from, say, a romantic perspective), and value-related (that is, related to social, political, moral, aesthetic, or religious values) ^23^. In addition to this, the humanities are particularly suitable for educating students to become well-informed, critical citizens who can reflect on socially urgent questions about life, health, education, justice, equality, liberty, etc. and participate fully in society and politics ^24^.

**5. *To serve society*.** Universities can serve society at a number of levels: local (a city or region), national, or international, humanity worldwide. What we have in mind here is serving society *epistemically*, that is, to help society acquire true belief, knowledge, and understanding about important issues. Of course, universities sometimes also serve society in a more practical manner, e.g., by way of proposing effective policies, producing medical interventions and other technologies. Such practical interventions are often based on scientific evidence, so the epistemic and the practical are not entirely separate, but they can be distinguished for analytical purposes. We focus on the former here, as our taxonomy concerns the *epistemic* rather than the *moral*, *practical*, or *social* responsibilities of universities.

Universities have the epistemic responsibility to serve society by, among other things, disseminating knowledge and understanding about issues that academics have investigated. Let us stress that we have knowledge *dissemination* rather than knowledge *utilization* in mind here, since we are concerned with epistemic rather than practical responsibilities of the university. So, what we have in mind are such things as press releases, expert advice, popular articles, opinion pieces, public lectures, interviews, and so on.

Serving society, however, is a two-way street. It’s not only about disseminating knowledge that has been produced by scientists, but also requires taking onboard insights from society and the general public. Universities can thus also serve society by taking in urgent questions, analyzing social concerns, and incorporating the perspectives and criticisms of non-scientists ^25^. Going even further, initiatives under the banner of ‘citizen science’ and ‘science with and for society’ promote the co-production of scientific knowledge by scientists and citizens ^26^. Such initiatives shouldn’t be thought of as replacing traditional science, but rather as complementing it in order to let science serve society better.

This is a responsibility for at least two reasons. First, many scientific and scholarly discoveries are so complex that if academics do not disseminate their knowledge, those discoveries will remain unknown among the larger audience. Second, it often requires extensive academic knowledge to understand the *importance* and *ramifications* of various discoveries. Knowledge and understanding are of intrinsic epistemic value. If the university does not serve society by sharing academic knowledge and understanding, then, for much academic knowledge and understanding, that value will be attained only by a very small group of academics in the relevant field. If the university takes its epistemic responsibility of knowledge dissemination seriously, then much larger groups – academics in other fields, society as a whole – will attain those epistemic values.

Finally, we should be clear that we don’t want to deny that the epistemic responsibilities we distinguish are related to each other in various ways. For instance, responsibility 1 (to foster research integrity) has to do with responsibility 2 (to teach for intellectual virtue): a university cannot be serious about research integrity but without teching for for curiosity, open-mindedness, thoroughness, and intellectual perseverance. The exact relations between these responsibilities is up for debate; we wanted to leave room for different opinions about this while working towards a potential consensus that these are indeed key epistemic responsibilities of universities. Even so, it is useful to draw analytic distinctions between the responsibilities. The way we formulated them makes it possible to check their presence relatively independently from one another and we consider that an advantage of our approach.

## Future steps

As indicated, we propose our normative taxonomy as a tool to assess the degree to which a university meets the Big Five epistemic responsibilities. Our proposal is a first attempt; in future work we aim to validate, test, amend, and implement the taxonomy. Particularly, we will work on distinguishing the five levels postulated for each epistemic responsibility more clearly and we will explore in more detail how our taxonomy differs from others and whether it can serve as a tool to rank universities. We envision doing this in four consecutive steps.

First, we want to fine-tune our taxonomy in a Delphi Study ^27, 28^ with international experts that aims in its first round at adding, replacing, and reformulating various epistemic responsibilities. The second and third Delphi rounds will seek consensus on the corresponding levels of meeting the responsibility at issue and explore what the best practices are in reaching higher levels of specific responsibilities.

Next, we will organize co-creation workshops ^29^ with representatives of relevant stakeholders in order to discuss a penultimate version of the taxonomy. The focus of the workshop will be on the operationalization of the levels of meeting the different epistemic responsibilities in a way which makes application of the taxonomy feasible, transparent, and as objective as possible.

Then, we will test and qualitatively evaluate the taxonomy in a number of universities, resulting in a definitive description of the responsibilities, the levels, and a tool-kit of best practices.

Finally, we will publish and disseminate the results on a dedicated website and explore whether the taxonomy is a suitable alternative for, or addition to, the currently dominant Academic Ranking of World Universities ^30^ and the Times Higher Education World University Rankings ^31^.

## Footnotes

^1^ Some of us have made this distinction before; see, for instance, Rik Peels, Jeroen de Ridder, Tamarinde Haven, Lex Bouter. (2019). “Value Pluralism in Research Integrity”, *Research Integrity and Peer Review* 4:18, https://rdcu.be/bPlwi

^2^ Brian Nosek et al. - Promoting an open research culture - Science 2015; 348 1422-5 en https://cos.io/our-services/top-guidelines/

^3^ For some of the outcomes, see Jeroen de Ridder, Rik Peels, René van Woudenberg. (2018). *Scientism: Prospects and Problems* (New York: Oxford University Press).

^4^ For discussion of these challenges, see, e.g.: Edwards, Marc, and Siddhartha Roy. (2017). “Academic research in the 21st century: Maintaining scientific integrity in a climate of perverse incentives and hypercompetition.” *Environmental Engineering Science* 34(1): 51–61; Haven, Tamarine, Lex Bouter, Yvo Smulders, and Joeri Tijdink. (2019). “Perceived publication pressure in Amsterdam: survey of all disciplinary fields and academic ranks.” *PLoS ONE* 14: e0217931, https://doi.org/10.1371/journal.pone.0217931; Anthony Kronman (2008). *Education’s End: Why Our Colleges and Universities Have Given Up on the Meaning of Life*. (New Haven, CT: Yale University Press); Martha Nussbaum. (2010). *Not for Profit: Why Democracy Needs the Humanities* (Princeton: Princeton University Press); Rosario Costa. (2019). “The Place of the Humanities in Today’s Knowledge Society.” *Palgrave Communications* 5, 38, https://doi.org/10.1057/s41599-019-0245-6; Daniel Greenberg. (2007). *Science for Sale: The Perils, Rewards, and Delusions of Campus Capitalism*. (Chicago, IL: University of Chicago Press); Hans Radder. (2010) *. The Commodification of Academic Research: Science and the Modern University.* (Pittsburgh, PA: University of Pittsburgh Press).

^5^ See National Academies of Sciences, Engineering, and Medicine. 2017. Fostering Integrity in Research. Washington, DC: The National Academies Press. https://doi.org/10.17226/21896

^6^ See, e.g., the Singapore Statement ( https://wcrif.org/guidance/singapore-statement); ALLEA (2017), *The European Code of Conduct for Research Integrity Revised Edition*, https://ec.europa.eu/research/participants/data/ref/h2020/other/hi/h2020-ethics_code-of-conduct_en.pdf, last visited May 14 ^th^ 2019.; DORA. (2018). San Francisco Declaration on Research Assessment, https://sfdora.org; VSNU (2018), *Netherlands Code of Conduct for Research Integrity, Revised Edition*, http://www.vsnu.nl/files/documents/Netherlands%20Code%20of%20Conduct%20for%20Research%20Integrity%202018.pdf, last visited May 14 ^th^ 2018.

^7^ See National Academies of Sciences, Engineering, and Medicine. 2017. Fostering Integrity in Research. Washington, DC: The National Academies Press. https://doi.org/10.17226/21896 and https://www.nap.edu/download/21896; and Hiney, Maura. (2015). “Research Integrity: What it Means, Why it Is important and How we Might Protect it”. EC Briefing Paper - Research Integrity ( https://www.scienceeurope.org/wp-content/uploads/2015/12/Briefing_Paper_Research_Integrity_web.pdf)

^8^ Fanelli - How many scientists fabricate and falsify research PLoS ONE 2009; 4(5); e5738. Note that these numbers concern scientists’ self-reporting; they go up considerably when researchers are asked whether they’re aware of colleagues having committed scientific fraud or engaged in questionable research practices.

^9^ KNAW Replication studies ( https://www.knaw.nl/nl/actueel/publicaties/replication-studies) and Baker, M (2016). “Is there a replicability crisis?” *Nature* 533 (7604): 452–454. Some of us have recently argued that replication is also a desideratum in the humanities. See Rik Peels, Lex Bouter. (2018). “The Possibility and Desirability for Replication in the Humanities”, *Palgrave Communications* 4:95, DOI: 10.1057/s41599-018-0149-x, reprinted in *Quantitative Methodologies: Novel Applications in the Humanities and Social Sciences*.

^10^ As some of us have argued, replicability is not always a desideratum in the humanities. See Rik Peels, Lex Bouter. (2018). “The Possibility and Desirability for Replication in the Humanities”, *Palgrave Communications* 4:95, DOI: 10.1057/s41599-018-0149-x; Rik Peels. (2019). “Replication and Replicability in the Humanities”, *Research Integrity and Peer Review* 4.2, https://doi.org/10.1186/s41073-018-0060-4.

^11^ Mark D. Wilkinson, Michael Dumontier, et al. 2016. “The FAIR Guiding Principles for scientific data management and stewardship.” *Scientific Data* 3: 160018. DOI: 10.1038/sdata.2016.18. And https://www.go-fair.org/fair-principles/

^12^ Brian A. Nosek, et al. (2018). “The preregistration revolution”. *PNAS* 115: 2600–2606.

^13^ David Moher, Florian Naudet et al. (2018). “Assessing scientists for hiring, promotion, and tenure”. PLoS Biol 16(3): e2004089. https://doi.org/10.1371/journal.pbio.2004089

^14^ See Heather Battaly, ed. (2010). *Virtue and Vice, Moral and Epistemic* (Oxford: Wiley-Blackwell); Baehr, Jason, ed. (2016). *Intellectual Virtues and Education: Essays in Applied Virtue Epistemology* (New York: Routledge).

^15^ See Ron Ritchhart. (2002). *Intellectual Character: What It Is, Why It Matters, and How to Get It* (San Francisco: Jossey-Bass).

^16^ See Robert Roberts and Jay Woods. (2007). *Intellectual Virtues* (New York: Oxford University Press); Jason Baehr. (2011). *The Inquiring Mind* (New York: Oxford University Press); Heather Battaly, ed. (2018). *Routledge Handbook of Virtue Epistemology* (London: Routledge).

^17^ Sylvia Berryman. (2016). “Ancient Atomism”, in: Edward N. Zalta (ed.), *The Stanford Encyclopedia of Philosophy* https://plato.stanford.edu/archives/win2016/entries/atomism-ancient/.

^18^ See William Shea, Mariano Artigas. (2003). *Galileo in Rome: The Rise and Fall of a Troublesome Genius*. Oxford: Oxford University Press; Remmert, Volker. (2005). “Galileo, God, and Mathematics." In T. Koetsier, L. Bergmans (eds.), *Mathematics and the Divine. A Historical Study.* Amsterdam: Elsevier, pp. 347–360. **

^19^ See Adrian Desmond, James Moore. (1992). *Darwin*. New York: W.W. Norton.

^20^ See Walter Isaacson. (2017). *Einstein. His Life and Universe*. New York: Simon & Schuster.

^21^ Thus also, Rik Peels. (2018). “Epistemic Values in the Humanities and the Sciences”, *History of Humanities* 3.1, 89–111.

^22^ See René van Woudenberg. (2018). “The Nature of the Humanities”, *Philosophy* 93: 109–140.

^23^ See chapter 2 of Richard Foley. (2018). *The Geography of Insight. The Sciences, the Humanities, How They Differ, Why They Matter*. Oxford: Oxford University Press.

^24^ See Martha Nussbaum. (2010). *Not for Profit: Why Democracy Needs the Humanities* (Princeton: Princeton University Press).

^25^ See Philip Kitcher (2011). *Science in a Democratic Society* (New York: Prometheus Books) or Hans Radder (2019). *From Commodification to the Common Good* (Pittsburgh, PA: University of Pittsburgh Press) for encompassing visions on how science can serve the needs of democratic societies.

^26^ See https://www.scienceforsociety.com and the European Commission’s SwafS-program: https://ec.europa.eu/research/swafs/index.cfm.

^27^ Terwee CB, Prinsen CAC, Chiarotto A, Westerman MJ, Patrick DL, Alonso J, Bouter LM, de Vet HCW, Mokkink LB. COSMIN methodology for evaluating the content validity of Patient-Reported Outcome Measures: a Delphi study. Quality of Life Research 2018; 27: 1159–70

^28^ https://web.njit.edu/~turoff/pubs/delphibook/delphibook.pdf.

^29^ Ramaswamya V, Ozcan K. What is co-creation? An interactional creation framework and its implications for value creation. Journal of Business Research Volume 84, March 2018, Pages 196–205 ( https://doi.org/10.1016/j.jbusres.2017.11.027)

^30^ http://www.shanghairanking.com/

^31^ https://www.timeshighereducation.com/world-university-rankings
