# Supplementary material for: Academia’s Big Five: a normative taxonomy for the epistemic responsibilities of universities
Source: F1000Res. 2020 Jul 6;8:862. Originally published 2019 Jun 13. [Version 2] doi: 10.12688/f1000research.19459.2 (PMC7934091; doi:10.12688/f1000research.19459.2)
Supplement: Peer review history

# Peer review: Reviewer response for version 2

**DOI:** 10.5256/f1000research.26802.r66478

**Published:** 2021-03-04

**Authors:** Britt Holbrook, Bart Penders

**Affiliations:** 1 Maastricht University, Maastricht, The Netherlands; 2 Department of Humanities, New Jersey Institute of Technology, Newark, NJ, USA

We appreciate the authors’ responses to our comments. Their changes to the manuscript manage to alleviate many of the concerns we have. However, one key concern – the Big One – remains: the authors' aspiration to act as rankers themselves. Even if the ranking they propose were conceptually and methodologically flawless– and we trust that the authors would not be willing to make such a bold claim to perfection, even after the extensive testing and validation they propose – the ranking would still result in moral and political problems associated with ranking. 

 In a wonderfully incisive Twitter thread (https://twitter.com/jelena3121/status/1356210115406397443), Jelena Brankovic, a long-time researcher on rankings, suggests that "Even if you made [rankings] super transparent, accountable, and well-governed, that could even aggravate the adverse effects." No ranking merely documents performance. The act of ranking necessarily inflates minor differences into different ranks. Every ranking is a zero sum game. One university can rise in the rankings only if another goes down. Insofar as every ranking is a zero sum game, every ranking generates reputation. Every ranking produces competition for that reputation. Indeed, reputational ranking becomes the One Big Goal for universities. The authors’ aspiration to turn the Big Five into a ranking will insure that, rather than working to improve their attention to their epistemic responsibilities, universities will treat those responsibilities merely as means to achieving a higher ranking. 

 We would like to invite the authors to frame their ambitions more cautiously. Used as a rubric for relatively low stakes formative assessment, the Big Five might well improve the performance of some universities. Deployed as a ranking for all universities, however, the Big Five will become all about the Big One.

Is the topic of the opinion article discussed accurately in the context of the current literature?

Partly

Are arguments sufficiently supported by evidence from the published literature?

Partly

Are all factual statements correct and adequately supported by citations?

Yes

Are the conclusions drawn balanced and justified on the basis of the presented arguments?

Yes

Reviewer Expertise:

NA

We confirm that we have read this submission and believe that we have an appropriate level of expertise to confirm that it is of an acceptable scientific standard.

# Peer review: Reviewer response for version 1

**DOI:** 10.5256/f1000research.21335.r57012

**Published:** 2019-12-11

**Authors:** Jennifer Byrne

**Affiliations:** 1 NSW Health Statewide Biobank, School of Medical Sciences, Faculty of Medicine and Health, The University of Sydney, Sydney, NSW, Australia

This is an interesting manuscript which is somewhat outside my field of expertise, which is centred around molecular biology and genetics. There is no doubt that this will have limited my ability to provide a detailed critique of the arguments presented, and this may also limit the value of some of my suggestions. Nonetheless I advance these in the hope that my comments will indicate how this article was received by someone outside the field, given that this paper will interest a broad range of academics and researchers.

 My main comments pertain to the taxonomy proposed in Table 1. I would not argue with the five epistemic responsibilities put forward; however, I did wonder whether five levels of taxonomy are required for any or all of the five proposed responsibilities. Some of the descriptions provided for levels I and II seem unlikely to occur in practice, and I could not always see clear distinctions between some levels proposed, most frequently for levels I and II.

 For example, for “1. To foster research integrity”, I could not see a clear difference between “Issues of detrimental research practices and responsible conduct of research are neglected” (level I) and “Issues…. are occasionally addressed” (level II). There may not be much difference in practice between something being “neglected” or “occasionally addressed”, and it is also not clear how “occasionally” would be reliably measured. Similarly, I could not see a clear difference between level III “Detrimental research practices are addressed… but there is no academic climate that actively stimulates responsible conduct of research” and level IV “There is an academic climate that detects and acts upon detrimental research practices and actively stimulates responsible conduct of research, but only at the level of individual researchers”.

 For “2. To teach for intellectual virtue”, I couldn’t see clear differences between level I “the university…. pays no attention to intellectual virtues” and level II “intellectual virtues are considered important, but not taught”. I also could not imagine any University that would fall into either of these categories.

 Again, I couldn’t see clear differences for the proposed levels I and II for “3. To address the big questions of life”, ie these questions being “neglected” (level I), versus “mentioned but discarded” (level II).

 For “4. To give humanistic inquiry and education a proper place”, I also saw no real distinction between the humanities being “marginalized” (level I) and “not marginalized but considered and treated as being inferior” (level II). Levels III, IV, and V commonly refer to humanities being given a “proper place” in the university, but this definition will naturally vary between universities according to their type, i.e. technical universities versus universities with a strong and stated focus upon the humanities. Some wording within levels III-V also lacked definition, i.e. “in isolation” (level III), “some other disciplines” (level IV), and “across disciplines” (level V). “Disciplines” can be defined quite narrowly by some universities, so working with other disciplines or across disciplines does not always imply working between the humanities and the sciences.

 Finally, for “5. To serve society”, universities where “research and teaching are confined to …purely academic challenges” (level I) could argue that they are serving society in this way, possibly by pursuing academic challenges that can indirectly linked to the big questions of life. It also seems unlikely that any university would be defined by level II “Research and teaching identify societal challenges, but the university leaves it to others to confer knowledge … relevant to those challenges”.

 Are all factual statements correct and adequately supported by citations?

  

1. Contemporary challenges such as hypercompetition, publication pressures, marginalisation of the humanities and commercialization of universities (page 3, paragraph 2) should be supported by references.
2. The status of reference 1 (footnote 1) which is a manuscript by Peels et al, could be updated (page 3).

 

 Are arguments sufficiently supported by evidence from the published literature?

 Although the manuscript states that “more transparency and specifically pre-registration of study protocols and data analysis plans will lead to considerable improvements (in the use of small sample sizes, selective reporting and other questionable research practices, from the preceding sentence), this seems to be somewhat of an overstatement. Firstly, more transparency and pre-registration may not impact on the use of small sample sizes, as these might represent feasibility constraints that pre-registration may not overcome. Secondly, I understand that there is not yet a substantial body of literature that describes the possible benefits and/or drawbacks of study pre-registration, by comparing the results and outcomes of pre-registered studies versus comparable studies that lacked pre-registration.

 Finally, the manuscript states that (the big questions of life) “are too important to be left entirely to non-academics”. This statement could be written in a more inclusive way, for example by indicating that as these questions are so important, they are everyone’s concern, including the concern of academics.

Is the topic of the opinion article discussed accurately in the context of the current literature?

Partly

Are arguments sufficiently supported by evidence from the published literature?

Partly

Are all factual statements correct and adequately supported by citations?

Partly

Are the conclusions drawn balanced and justified on the basis of the presented arguments?

Yes

Reviewer Expertise:

Molecular biology, molecular genetics, human tissue biobanking, research integrity

I confirm that I have read this submission and believe that I have an appropriate level of expertise to confirm that it is of an acceptable scientific standard.

# Response

**Published:** 2020-07-02

**Authors:** Rik Peels

**Affiliations:** Vrije Universiteit Amsterdam, Denmark

Jennifer Byrne (JB) first notes that the distinctions between the different levels of meeting our responsibilities remain somewhat unclear. This is a good and important point; these levels ought to be distinguished more clearly. In fact, this is one of the main goals of our new project entitled *Epistemic Progress in the University (2020-2023) *that was recently funded by the Templeton World Charity Foundation. JB’s remarks provide plenty of food for thought on the distinctiveness of the levels as we formulated them. We are very grateful for that and will certainly take these suggestions into account while developing the taxonomy further. We now mention the new project in the introduction and state that we need to do more work on the distinctiveness of the five levels postulated for each epistemic responsibility.

 Second, we have updated the publication status of footnote 1 and added references to substantiate what we say on hypercompetition, publication pressure, marginalization of the humanities, and commercialization of universities. 

 Third, JB thinks that we overstate the benefits of pre-registration. We partly disagree. We wrote: “Although these phenomena are understudied, it is clear that more transparency and specifically preregistration of study protocols and data analysis plans will lead to considerable improvements.” We didn’t consider this to be a particularly strong claim, but we have now changed it into: “Although these phenomena are understudied, it is to be expected that more transparency and specifically preregistration of study protocols and data analysis plans will lead to improvements.” 

 Finally, we have adopted JB’s suggestion for a more inclusive formulation about whose concern the big questions are. The text now reads: “The big questions are everyone’s concern, academics included.”

# Peer review: Reviewer response for version 1

**DOI:** 10.5256/f1000research.21335.r49894

**Published:** 2019-07-01

**Authors:** Britt Holbrook, Bart Penders

**Affiliations:** 1 Department of Humanities, New Jersey Institute of Technology, Newark, NJ, USA; 2 Maastricht University, Maastricht, The Netherlands

This article proposes what Peels *et al.* call a “normative taxonomy” of the “Big Five” epistemic responsibilities universities ought to meet: (1) fostering research integrity, (2) teaching (for) intellectual virtues, (3) addressing the big questions, (4) valuing the humanities, and (5) serving society. This normative taxonomy essentially serves as an *analytical rubric* designed to assess the performance of universities. One could easily imagine – and in fact, Peels *et al.* propose as much in §3 – refining the rubric presented in Table 1, using it to assess the performance of various universities, and then comparing their “Big Five” scores to their world university rankings. We agree that undertaking these tasks presents many enticing possibilities, especially to those of us inclined against bean counting approaches to accountability. Nevertheless, since they position themselves as aspiring university rankers, they should address the range of critiques targeting existing rankings, their methodologies, and the performativity of these rankings. They should then situate their own approach within that context, arguing especially that it is an improvement on – and escapes the criticisms levelled at – existing rankings. This is our global concern.

 Below, we offer a number of other substantial concerns, followed by a shorter list of minor issues to consider:

**Why these five?**

 Peels *et al. *propose five epistemic responsibilities of universities as *the *Big Five. The authors point out that they consider each of the Big Five equally important. They also defend each of the five as indeed responsibilities that universities ought to meet. However, they fail to argue that these five epistemic responsibilities are the most important epistemic responsibilities for universities, that these five are in fact equally important, that these five are sufficient to capture the epistemic responsibilities universities ought to meet, or that universities have no other equally worthy responsibilities that ought to be included.

**Why five?**

 We agree that the proposed Big Five are epistemic responsibilities that universities ought to meet. Using the proposed rubric to evaluate universities might provide valuable information about particular areas of strength and weakness at a given university. However, designing the rubric around five separate epistemic responsibilities suggests that universities could meet each of their epistemic responsibilities to varying degrees. One university might be particularly strong in terms of fostering research integrity but fail miserably at teaching for intellectual virtue. Another might excel at addressing the big questions but do a lousy job of valuing the humanities. Although these possibilities sound *prima facie* plausible, using an analytic approach to epistemic responsibilities also generates some less than desirable outcomes. Could a university that fails to teach for intellectual virtue really excel at fostering research integrity? That seems plausible only if we have a very limited procedural view of what counts as fostering research integrity. Without valuing the humanities, how could a university excel at addressing the big questions? Unless we adopt scientism, the answer seems to be that it could not. Similar problems arise when we consider the relations between the other proposed epistemic responsibilities. The use of a taxonomical vocabulary further strengthens this worry. Taxonomies should display not only differences, but also relationships and similarities. The current form of representation is not, in that sense, a taxonomy, but (for the most part) a normative *list*. Only when the ties binding the elements together are conceptualised convincingly can we convincingly speak of a taxonomy. For this reason, we suggest that the authors consider enriching their list to raise it to the level of a taxonomy or abandon talk of a ‘taxonomy’ altogether. The authors could adopt the vocabulary of an analytical rubric, instead.

 Even if the authors decide to use the language of rubrics rather than taxonomies, we suggest that the authors consider a more holistic approach along with an analytic one, just as virtue ethics appeals not only to the individual virtues, but also to the question of character. Rather than describing teaching for intellectual virtue as part of *Bildung*, perhaps the authors could consider fostering the ability to pursue *Wissenschaft* while cultivating *Bildung *as analogous to a university’s character. Call it the *W-B *rubric, under which different (now, no longer separate) epistemic responsibilities could be grouped together to indicate different levels of overall achievement. In order for a university to score best on the *W-B *rubric, it would have to excel at meeting *all *of its epistemic responsibilities (for a brief discussion of the difference between analytic and holistic rubrics, as well as examples and further references, see *Rubrics: useful assessment tools. Centre for Teaching Excellence, University of Waterloo* ^1^).

**Why insist on separating epistemic from other responsibilities universities have?**

 This question follows on the previous one, in that it aims to nudge the authors away from using only an analytic approach. By insisting on distinguishing epistemic responsibilities from moral, practical, and social responsibilities, the authors impoverish the idea of what it means for universities to serve society. If the goal is to work towards alternative forms of assessment and accountability (thereby politicizing them further), excluding other responsibilities and artificially fragmenting the landscape accordingly, one would expect [a] the epistemic responsibility taxonomy to be situated within a larger responsibility taxonomy and/or [b] an argument that leaving other responsibilities aside is preferable. More in line with the suggestion to pursue a more holistic approach, we must ask: could we really say that a university is a good university if it meets only its epistemic responsibilities? We think not. Perhaps the *W-B *rubric we suggest, or another holistic approach, could help address this issue. Note that one could also include analytical rubrics for moral, practical, and social responsibilities of universities and incorporate the responsibilities included in each of these separate rubrics into a holistic approach.

 Our point is not that analysis is always bad. Indeed, analysis can be quite informative and useful as part of a formative evaluation. We are simply suggesting that the authors consider using their analytic approach to complement a more holistic approach.

**Who counts, and why do they count?**

 In §3, the authors suggest optimising their rubric by means of a Delphi Study “with international experts”, which could lead to fine-tuning it, but possibly also to radical revisions. If the latter is the case, then what is the status of the current five epistemic responsibilities? When it comes to the actual Delphi study, whom they choose to participate in the study will likely make a very big difference to the final design of the rubric. Presumably, the authors focus on “experts” because they assume experts know more about epistemic responsibilities than non-experts do. Yet, the way in which the authors present it suggests a retreat to the deficit/diffusion model of public understanding of science. Not only has the deficit model been shown to be factually incorrect, it also presupposes a social contract for science and scholarship that imagines universities as ivory towers. In their discussion of a university’s responsibility to serve society epistemically, the authors focus on the supply side of knowledge production, suggesting that knowledge dissemination is how best to serve society. Arguably, however, serving society – even if we limit this to an epistemic responsibility – means something other than telling society what we academics think they need to know. Here again, the separation between epistemic and other responsibilities creates a lot of friction, since a departure from the deficit model requires a high degree of interaction and participation beyond universities. In fact, one could argue that interaction and participation are epistemic requirements (co production of knowledge).

 Along these lines, we suggest that non-experts may have valuable feedback to offer, even if the authors ultimately decide only to pursue the development of an analytic rubric for epistemic responsibilities of universities. One way to include the demand side of knowledge production in the design of their rubric would be to recruit non-expert stakeholders to participate in the proposed Delphi Study and workshops or organise parallel expert (Delphi) and citizen (Citizen Summit) consultations.

**We also have a few more minor points that nevertheless warrant attention:**

1.  Despite the argument that the rubric is to be applied to entire universities, the responsibilities focus very much on individuals and groups (which seem to be multiple individuals in the ways in which they are discussed) and less on the level of structures and collectives.
2. Peels *et al.* frame irreproducibility solely as the result of sub-par science and thus as a research integrity issue. Their previous work, as well current scholarly debates on the characters and qualities or irreproducibiluty and irreplicability, takes up a much more nuanced position. Perhaps the authors will consider adding some of that nuance here.
3. Excusing a few universities (technical universities or polytechnics, in this case) of taking responsibility for one of the epistemic responsibilities (#4) suggests that giving humanistic inquiry and education a proper place is optional. This seems to conflict with the idea that all responsibilities are equally important. It also opens up possibilities for policies that deprioritise humanities research and teaching (cf. the current *Van Rijn* report in NL).

Is the topic of the opinion article discussed accurately in the context of the current literature?

Partly

Are arguments sufficiently supported by evidence from the published literature?

Partly

Are all factual statements correct and adequately supported by citations?

Yes

Are the conclusions drawn balanced and justified on the basis of the presented arguments?

Yes

Reviewer Expertise:

NA

We confirm that we have read this submission and believe that we have an appropriate level of expertise to confirm that it is of an acceptable scientific standard, however we have significant reservations, as outlined above.

## References

[1] Rubrics: Useful Assessment Tools. *Centre for Teaching Excellence, University of Waterloo*.2019; Reference source

# Response

**Published:** 2020-07-02

**Authors:** Rik Peels

**Affiliations:** Vrije Universiteit Amsterdam, Denmark

We thank the reviewers for their helpful comments and suggestions. Below, we reply to them.

*Relation to Other Rankings*

 Holbrook and Penders (H&P) urge that we do two things. First, that we situate our taxonomy in the wider context of other rankings. Second, that we address criticisms of existing rankings, and explain why we think ours is an improvement.

 H&P are right that there are many other rankings, even many other non-standard rankings that are not merely cashed out in terms of publications. Yet, many of them are based on quantitative data, such as (i) number of students; (ii) granted research proposals; (iii) Nobel prize winners; (iv) citation indices; (v) the result of surveys on the opinions on stakeholders with respect to e.g. the reputation of universities. Our proposal focuses on epistemic responsibilities. Whereas other rankings are mainly based on quantitative data and opinions of stakeholders, the instrument we propose is based on mainly qualitative data, and will lead to an ordering in *normative* categories. Because of these differences, it isn’t immediately clear that extant criticisms of other rankings would apply to our proposed taxonomy. But we’ll look into this more systemically in future work as part of a new project on Epistemic Progress in the University (also funded by the Templeton World Charity Foundation). We explain this in the introduction and the section on Future steps.

*Why These Five?*

 H&P note that we don’t argue (a) that our five are the most important areas of responsibility; (b) that they are equally important; (c) that they are *sufficient* to capture the epistemic responsibilities of universities; (d) that universities have no other equally worthy responsibilities.

 As to (a)–(c), H&P are right that more support is needed to back up these claims – the current paper lays out a proposal based on a survey of literature about epistemic responsibilities of the university and our own analysis thereof. But providing further support is exactly what we aim to do in follow-up work that’s part of the abovementioned new project.

 As to (d), universities indeed have other, equally important responsibilities. Among them are moral responsibilities (like providing safe environments for students and faculty, and taking care of the wellbeing of human and animal test subjects, treating indigenous people fairly); legal responsibilities; financial responsibilities; social responsibilities (like producing useful technologies and effective medical interventions), and more. What sets universities apart from many other organizations and institutions is that their main goals are *epistemic* in nature: their primary aims are to produce and disseminate knowledge and understanding. That is why we focus on these. We now make this more explicit in the introduction.

*The Relations between Various Values*

 H&P say that the values are interrelated (if there is research integrity, there will or must be such a thing as teaching virtue; and how can universities address the Big Questions without cultivating the humanities?) and that we do not acknowledge this fact. And this, they maintain, undermines our goal of offering a taxonomy—for a taxonomy “should display not only differences but also relationships and similarities”. Instead of offering a taxonomy, they urge, we could also offer *rubrics*.

             We agree that the epistemic responsibilities we distinguish are related to each other. For instance, responsibility 1 (to foster research integrity) surely has to do with responsibility 2 (to teach for intellectual virtue): it would be highly surprising if a university is serious about research integrity but does not teach for curiosity, open-mindedness, thoroughness, and intellectual perseverance. However, this doesn’t mean that we cannot draw analytic distinctions between the responsibilities that allow us to focus on different parts and aspects of universities’ epistemic responsibilities. It may not always be possible (or even desirable) in practice to implement these responsibilities through separate activities, interventions, or policies, but they can be distinguished through an analytical lens. We consider that an advantage of our approach. Furthermore, the exact relations between these responsibilities is up for debate, so we wanted to leave room for different opinions about the relations between these responsibilities while working towards more consensus *that* these are indeed key epistemic responsibilities of universities. We have added this reasoning at the end of section 2.

             As to the suggestion of explicating relationships and dependencies and developing a rubric rather than a taxonomy, we are happy to take these ideas into consideration as we continue the development of this proposal in future work.

*A More Holistic Rubric?*

 H&P add that just as the virtues of an individual person are expressions of that person’s *character*, so the university has (or should have) a *character*, and their friendly suggestion is that the character of the university is *To pursue Wissenschaft while cultivating Bildung*.

 We are sympathetic to this suggestion; universities can have relatively stable dispositions to pursue particular values reliably. We might call this the university’s character or, alternatively, its organizational culture. We take it, though, that this suggestion is already covered by our proposal. It is an epistemic responsibility of universities to give the humanities a proper place next to the natural and social sciences. Our focus on intellectual virtues, moreover, is very much in line with the ideal of *Bildung*.

*Deficit/diffusion Model of Public Understanding*

 H&P say that we rely on the incorrect deficit/diffusion model of science communication: the public has a deficit of knowledge, and science communication aims to diffuse the deficit by providing knowledge.

             We certainly didn’t mean to assume this model, although we can see how our focus on knowledge dissemination under the epistemic responsibility of serving society might give the impression that the role of universities merely is to better inform the public. To prevent this misunderstanding, we have added a new paragraph where we explicitly state that the responsibility of serving society can be a two-way street, where citizens can offer valuable input on which research questions to pursue, how to best pursue them, and how to communicate and use research findings. In some cases, the production of scientific knowledge can even amount to co-creation between scientists, stakeholders, and citizens.

             The suggestion to include citizens and various stakeholders in proposed Delphi studies is a valuable one, that we will take onboard.

*Smaller Points*

 H&P raise three smaller points:

1. We focus, they say, more on individuals than on structures and collectives. We’re not sure what gave rise to this impression – as we think of them, all five responsibilities really are responsibilities of the university as a whole, so both its leadership and the individuals affiliated with it, as well as its organizational structure, policies, incentives, etc. We are keenly aware that meeting these responsibilities is as much an issue of individual action as it is of collective action, formal and informal organizational structures and the incentives they give rise to. And, in fact, even broader structures and systems in society. In developing our proposal further, however, we will make sure to keep both the individual and the organizational level in focus.
2. In this paper we suggest that irreproducibility is a sign of sub-par science, so H&P say, and they suggest that we include in a further version of our paper our more nuanced (and already published) views. We’ve done this in the revised version of the paper (note 9).
3. We make it seem as if the humanities are *optional*. This may be a misunderstanding. All we wanted to do is acknowledge the existence of technical universities, which may not need fully-fledged humanities departments. We do emphasize that they too have responsibilities in addressing the big questions.
